# MicroRNAs of Epstein-Barr Virus Attenuate T-Cell-Mediated Immune Control *In Vivo*

**DOI:** 10.1128/mBio.01941-18

**Published:** 2019-01-15

**Authors:** Anita Murer, Julia Rühl, Andrea Zbinden, Riccarda Capaul, Wolfgang Hammerschmidt, Obinna Chijioke, Christian Münz

**Affiliations:** a Viral Immunobiology, Institute of Experimental Immunology, University of Zürich, Zürich, Switzerland; b Institute of Medical Virology, University of Zürich, Zürich, Switzerland; c Research Unit Gene Vectors, Helmholtz Zentrum München, German Research Center for Environmental Health and German Centre for Infection Research (DZIF), Partner Site Munich, Munich, Germany; d Institute of Pathology and Medical Genetics, University Hospital Basel, Basel, Switzerland; Icahn School of Medicine at Mount Sinai

**Keywords:** Epstein-Barr virus, cytotoxic T cells, humanized mice, immune escape, lymphoma, miRNA

## Abstract

The human persistent and oncogenic Epstein-Barr virus (EBV) was one of the first viruses that were described to express viral microRNAs (miRNAs). These have been proposed to modulate many host and viral functions, but their predominant role *in vivo* has remained unclear. We compared recombinant EBVs expressing or lacking miRNAs during *in vivo* infection of mice with reconstituted human immune system components and found that miRNA-deficient EBV replicates to lower viral titers with decreased frequencies of proliferating EBV-infected B cells. In response, activated cytotoxic EBV-specific T cells expand to lower frequencies than during infection with miRNA-expressing EBV. However, when we depleted CD8^+^ T cells the miRNA-deficient virus reached similar viral loads as wild-type EBV, increasing by more than 200-fold in the spleens of infected animals. Furthermore, CD8^+^ T cell depletion resulted in lymphoma formation in the majority of animals after miRNA-deficient EBV infection, while no tumors emerged when CD8^+^ T cells were present. Thus, miRNAs mainly serve the purpose of immune evasion from T cells *in vivo* and could become a therapeutic target to render EBV-associated malignancies more immunogenic.

## Abstract (precis)

Epstein-Barr virus (EBV) infects the majority of the human population and usually persists asymptomatically within its host. Nevertheless, EBV is the causative agent for infectious mononucleosis (IM) and for lymphoproliferative disorders, including Burkitt and Hodgkin lymphomas. The immune system of the infected host is thought to prevent tumor formation in healthy virus carriers. EBV was one of the first viruses described to express miRNAs, and many host and viral targets were identified for these *in vitro*. However, their role during EBV infection *in vivo* remained unclear. This work is the first to describe that EBV miRNAs mainly increase viremia and virus-associated lymphomas through dampening antigen recognition by adaptive immune responses in mice with reconstituted immune responses. Currently, there is no prophylactic or therapeutic treatment to restrict IM or EBV-associated malignancies; thus, targeting EBV miRNAs could promote immune responses and limit EBV-associated pathologies.

## INTRODUCTION

Epstein-Barr virus (EBV) is a ubiquitous human gammaherpesvirus that is carried by the majority of the adult human population as an asymptomatic but persistent infection (1). At the same time, it is one of the most growth-transforming infectious agents and the only one that can readily immortalize its main host cell, the human B cell, in culture (2). Accordingly, EBV is associated with lymphomas and epithelial cell cancers, which express different subsets of latent EBV gene products. Diffuse large B cell lymphomas and *in vitro*-transformed lymphoblastoid cell lines (LCLs) are positive for six EBV nuclear antigens (EBNAs), two latent membrane proteins (LMPs), two nontranslated Epstein-Barr virus-expressed RNAs (EBERs), and two clusters of microRNAs (miRNAs), the smaller BHRF1 miRNA cluster (3 pre-miRNAs) and the larger BART miRNA cluster (22 pre-miRNAs), giving rise to at least 44 mature miRNAs (3). Only one EBNA (EBNA1) and the two LMPs can be found in classical Hodgkin lymphoma and nasopharyngeal carcinoma, but all nontranslated RNAs are expressed. Finally, Burkitt lymphoma and EBV-associated gastric carcinoma further downregulate latent EBV protein expression and are positive only for EBNA1 but continue to express the nontranslated RNAs. These expression patterns in EBV-associated malignancies are mirrored in B cell differentiation stages of healthy EBV carriers (4). In quiescent memory B cells, EBV protein expression is abolished altogether and only nontranslated EBV RNAs are expressed. This documents the importance of EBERs and viral miRNAs for all latent and growth-transforming stages of EBV infection.

Fortunately, even though all premalignant EBV infection patterns are present in healthy EBV carriers, only a very small fraction of infected individuals develop virus-associated tumors, with an estimated incidence rate of 200,000 new cases of EBV-associated malignancies annually (5). It is thought that the immune system prevents EBV-driven tumor development, because EBV-associated lymphomas occur at increased frequencies in primary and acquired immunodeficiencies (6, 7). Acquired immunodeficiency due to HIV coinfection and primary immunodeficiencies due to mutations in the cytotoxic machinery, lymphocyte costimulation, and T cell receptor signaling identify CD8^+^ T cells as the crucial immune compartments for the control of EBV infection and prevention of associated lymphomagenesis. Accordingly, some EBV-associated tumors can be cured by adoptive transfer of EBV-specific T cell lines (8). Furthermore, in preclinical *in vivo* models of persistent EBV infection, utilizing mice with reconstituted human immune system components (huNSG mice), T cell depletion leads to increased viral loads and lymphoma formation (9–11). EBV seems to strike the right balance, ensuring its persistence after primary infection and allowing sufficient immune control to protect its host. Therefore, it is perhaps not surprising that it has been found *in vitro* that EBV-expressed miRNAs also regulate this T-cell-mediated immune control and dampen antigen presentation on major histocompatibility complex (MHC) class I and II molecules to CD8^+^ and CD4^+^ T cells, respectively (12, 13). However, the importance of this immune evasion by EBV-contained miRNAs remains unclear *in vivo*.

In order to address this question, we explored recombinant Epstein-Barr viruses, lacking either the larger BART cluster of miRNAs or all EBV-expressed miRNAs. In the absence of all EBV miRNAs, the virus established much lower viral titers after infection of huNSG mice. Surprisingly, CD8^+^ T cell depletion restored EBV infection to titers comparable to wild-type virus infection, causing an increase in viral loads by more than 200-fold in the spleen and lymphoma formation in the majority of infected animals. Our findings suggest that EBV-expressed miRNAs mainly serve the purpose of compromising T-cell-mediated immune control and highlight the importance of this immune control in the prevention of EBV-associated lymphoma formation.

## RESULTS

### EBV infection is attenuated in the absence of viral miRNAs.

**FIG 1:**
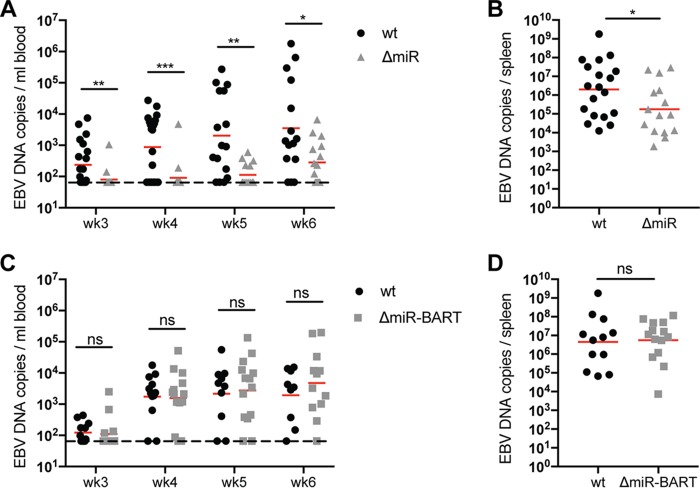
EBV infection is attenuated in the absence of viral miRNAs. (A and C) Blood DNA viral loads over time as determined by qPCR of huNSG mice infected with either wt, ΔmiR (A), or ΔmiR-BART (C) EBV for 5 to 6 weeks (*n* = 14 to 21/group). The horizontal dashed line indicates the lower limit of quantification (LLOQ). Values below the LLOQ were raised to the LLOQ and plotted on the LLOQ line. (B and D) Splenic endpoint viral DNA loads as determined by qPCR of huNSG mice infected with either wt, ΔmiR (B), or ΔmiR-BART (D) EBV for 5 to 6 weeks (*n* = 12 to 16/group). (A to D) Pooled data from 4 wt and ΔmiR-BART and 6 wt and ΔmiR experiments are displayed with geometric mean. *, *P* ≤ 0.05; **, *P* ≤ 0.01; ***, *P* ≤ 0.001, Mann-Whitney U test.

EBV devoid of its BHRF1 miRNA locus has previously been shown to delay systemic infection in a humanized mouse model without altering the tumor formation capacity of EBV (14). To further investigate the role of EBV miRNA *in vivo*, we used recombinant EBV strains that lack all EBV miRNAs (ΔmiR) (15) or only BART miRNAs (ΔmiR-BART) to infect NOD-*scid* γ_c_^null^ mice with reconstituted human immune system compartments (huNSG mice). Our group and others have previously shown that the huNSG mouse model is a suitable model for EBV infection and cell-mediated immune control *in vivo* (9–11, 16–19). In order to determine the pathogenic potential of ΔmiR and ΔmiR-BART EBV, we inoculated huNSG mice with 10^5^ Raji-infectious units (RIU) of the respective viruses and monitored infection compared to wild-type (wt) EBV for 5 to 6 weeks. The viral DNA burden was significantly lower in mice infected with ΔmiR than with wt EBV, but comparable between ΔmiR-BART and wt EBV over the entire observation period in blood, starting at 3 weeks after infection when viral loads became reliably detectable for the first time (Fig. 1A and C), and at the end of the experiments in spleen (Fig. 1B and D). Hence, these data suggest that ΔmiR EBV has a reduced, whereas ΔmiR-BART EBV has a similar, infectious capacity compared to wt EBV.

### Reduced frequencies of proliferating EBV-infected cells in the absence of viral miRNAs.

**FIG 2:**
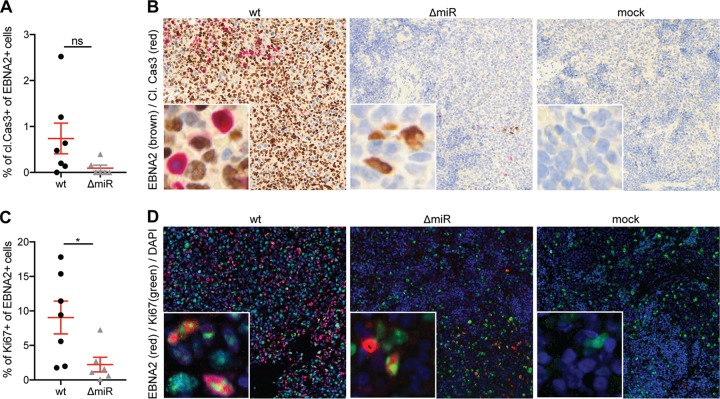
Reduced proliferation of EBV-infected cells in the absence of viral miRNAs. (A and B) Quantification of the frequency of cleaved caspase 3^+^ (cl.Cas3) EBNA2^+^ cells of all EBNA2^+^ cells (*n* = 6 to 7/group) (A) and representative immunohistochemistry for EBNA2 (brown) and cl.Cas3 (red) (original magnification, ×200) in splenic sections of huNSG mice infected with ΔmiR or wt EBV 5 to 6 weeks p.i. or noninfected mice (mock) (B). (C and D) Quantification of the frequency of Ki67^+^ EBNA2^+^ cells of all EBNA2^+^ cells (*n* = 6 to 7/group) (C) and representative immunofluorescence for EBNA2 (red), Ki67 (green), and DAPI (blue) (original magnification, ×200) in splenic sections of huNSG mice infected with ΔmiR or wt EBV 5 to 6 weeks p.i. or mock (D). (A and C) Pooled data from 3 experiments with mean ± SEM (unpaired *t* test with Welch’s correction).

Next, we investigated if the reduced titers of EBV in the absence of its miRNAs are due to decreased proliferation or increased apoptosis. Primary B cells infected with EBV lacking BHRF1 miRNAs were shown to undergo increased apoptotic death and decreased proliferation during the early infection period *in vitro* (15, 20). We therefore examined the frequency of proliferating and apoptotic cells in EBV-infected cells in our *in vivo* system using splenic sections of wt and ΔmiR EBV-infected mice. Immunohistochemical analysis of costaining for cleaved caspase 3 (cl. Cas3) and the viral protein EBNA2 suggested that there was less apoptotic activity in ΔmiR-infected cells than in wt-infected cells, although this difference did not reach statistical significance (Fig. 2A and B). Overall, the level of cl. Cas3^+^ EBNA2^+^ cells was very low (Fig. 2A). Immunofluorescence costaining for Ki67 and EBNA2 revealed a significantly higher frequency of proliferating EBNA2-positive cells in wt- than in ΔmiR-infected mice (Fig. 2C and D). However, established LCLs generated *in vitro* with either wt or ΔmiR EBV did not show a growth difference when quantifying total cell numbers over 12 consecutive days (see Fig. S1 in the supplemental material). These results indicate that reduced viral titers in the absence of EBV miRNA might be due to reduced proliferation of infected cells or other factors, such as increased immune control of proliferating infected cells.

10.1128/mBio.01941-18.1 FIG S1 Similar growth of established wt or ΔmiR LCLs. Absolute numbers of wt and ΔmiR LCLs over 12 consecutive days were determined by trypan blue staining (4 autologous pairs from different donors). Download FIG S1, TIF file, 0.1 MB. Copyright © 2019 Murer et al. 2019 Murer et al. This content is distributed under the terms of the Creative Commons Attribution 4.0 International license.

### Activation and memory formation of CD8^+^ T cells correlate with EBV viral load.

**FIG 3:**
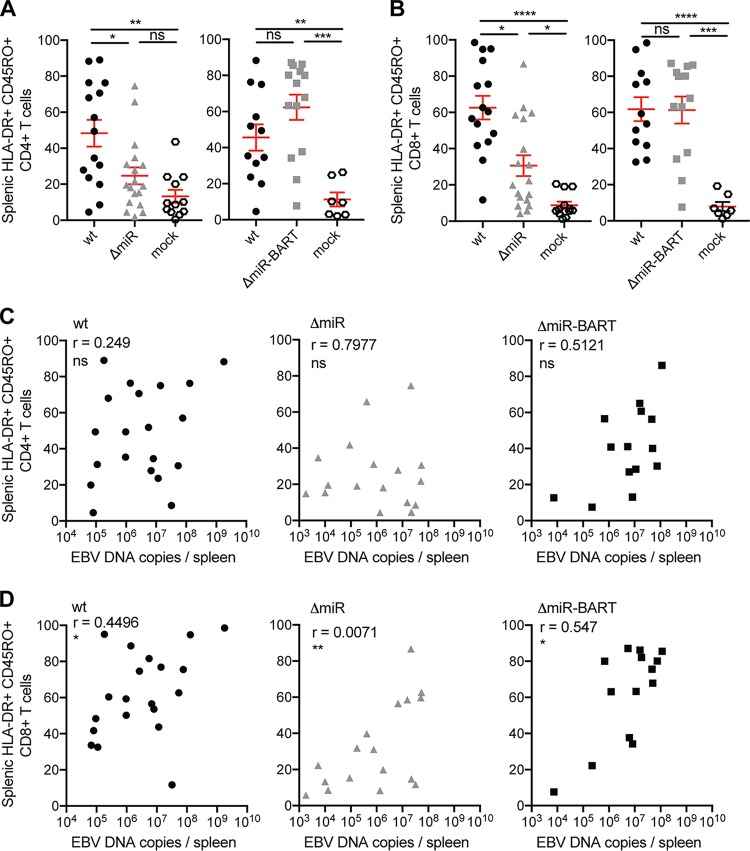
Activation and memory formation of CD8^+^ T cells correlate with EBV viral load. (A and B) The frequency of splenic HLA-DR^+^ CD45RO^+^ CD4^+^ T cells (A) and splenic HLA-DR^+^ CD45RO^+^ CD8^+^ T cells (B) of huNSG mice infected with either 10^5^ RIU of wt, ΔmiR, or ΔmiR-BART EBV 5 to 7 weeks p.i. or mock huNSG mice (*n* = 7 to 18/group) was determined by flow cytometry. (C and D) Correlation of the frequencies of activated memory CD4^+^ (C) and activated memory CD8^+^ (D) T cells, from panels A and C, respectively, with the splenic endpoint viral DNA loads as determined by qPCR for each infected group. (A and B) Pooled data from 4 wt and ΔmiR-BART and 5 wt and ΔmiR experiments with mean ± SEM. *, *P* ≤ 0.05; **, *P* ≤ 0.01; ***, *P* ≤ 0.001; ****, *P* ≤ 0.0001, Mann-Whitney U test. (C and D) Pooled data from 4 to 7 experiments. *, *P* ≤ 0.05; **, *P* ≤ 0.01, Spearman correlation.

Infection of huNSG mice with wt EBV has been reported to induce the activation and expansion of human CD8^+^ T cells in huNSG mice (10, 19). Since EBV miRNAs control adaptive antiviral immunity *in vitro* (12, 13), we assessed T cell activation and memory formation upon infection with ΔmiR, ΔmiR-BART, and wt EBV *in vivo*. In line with the EBV DNA burden in blood and spleen (Fig. 1), we detected similar frequencies of HLA-DR^+^ CD45RO^+^ CD4^+^ T cells (Fig. 3A and Fig. S2A, right panels) and HLA-DR^+^ CD45RO^+^ CD8^+^ T cells (Fig. 3B and Fig. S2B, right panels) in mice infected with wt and ΔmiR-BART EBV, whereas mice infected with ΔmiR EBV showed reduced frequencies compared to wt EBV 5 to 6 weeks postinfection (p.i.) (Fig. 3A and B, left panels, and Fig. S2A and B, left panels). Investigating specifically the formation of effector or central memory CD8^+^ T cells, frequencies of blood and splenic effector memory (CCR7^−^ CD45RA^−^) CD8^+^ T cells were increased in wt- as well as ΔmiR-infected animals compared to noninfected huNSG mice; however, in the absence of EBV miRNAs these frequencies were significantly lower than wt infection (Fig. S3A and C). CCR7^+^ CD45RA^−^ central memory CD8^+^ T cells were increased only in the spleen of wt EBV-infected compared to noninfected mice (Fig. S3B) but not in the blood of wt EBV-infected animals nor in either spleen or blood of ΔmiR EBV-infected animals (Fig. S3B and D). The frequency of HLA-DR^+^ CD45RO^+^ CD4^+^ T cells in both blood and spleen did not correlate with EBV DNA loads in the matching organ for any of the infected groups, except in blood for wt-infected mice (Fig. S3C and S2C). In contrast, the frequency of HLA-DR^+^ CD45RO^+^ CD8^+^ T cells in spleen and splenic EBV DNA load correlated significantly for all infection groups (Fig. 3D), while this correlation was absent in the blood (Fig. S2D). This suggests that EBV viral loads primarily drive activated CD8^+^ T cell expansions. Therefore, the observed activation and memory formation of CD8^+^ T cells seem to correlate with EBV DNA burden, which might indicate improved immune control by the less expanded CD8^+^ T cells during ΔmiR infection or decreased CD8^+^ T cell expansion due to lower antigenic load.

10.1128/mBio.01941-18.2 FIG S2 Activation and memory formation of CD8^+^ T cells correlated with EBV viral load. (A and B) The frequency of blood HLA-DR^+^ CD45RO^+^ CD4^+^ T cells (A) and blood HLA-DR^+^ CD45RO^+^ CD8^+^ T cells (B) and of huNSG mice infected with either wt, ΔmiR, or ΔmiR-BART EBV 5 to 6 weeks p.i. or noninfected control (mock) huNSG mice (*n* = 7 to 16/group) was determined by flow cytometry. (C and D) Correlation of the frequencies of activated memory CD4^+^ (C) and activated memory CD8^+^ (D) T cells, from panels A and C, respectively, with the blood endpoint viral DNA loads as determined by qPCR for each infected group. (A and B) Pooled data from 4 wt and ΔmiR-BART and 5 wt and ΔmiR experiments with mean ± SEM. *, *P* ≤ 0.05; **, *P* ≤ 0.01; ***, *P* ≤ 0.001; ****, *P* ≤ 0.0001, Mann-Whitney U test. (C and D) Pooled data from 4 to 7 experiments. *, *P* ≤ 0.05; **, *P* ≤ 0.01, Spearman correlation. Download FIG S2, TIF file, 1.0 MB. Copyright © 2019 Murer et al. 2019 Murer et al. This content is distributed under the terms of the Creative Commons Attribution 4.0 International license.

10.1128/mBio.01941-18.3 FIG S3 Primarily effector memory CD4^+^ and CD8^+^ T cells are generated in the absence or presence of EBV miRNAs. The frequency of blood (A) or splenic (C) effector memory CCR7^−^ CD45RA^−^ CD8^+^ T cells and blood (B) or splenic (D) central memory CCR7^+^ CD45RA^−^ CD8^+^ T cells of huNSG mice infected with either wt or ΔmiR EBV 5 to 6 weeks p.i. or noninfected control (mock) huNSG mice (*n* = 11 to 12/group) was determined by flow cytometry. (A to D) Pooled data from 4 experiments with mean ± SEM. *, *P* ≤ 0.05; **, *P* ≤ 0.01; ****, *P* ≤ 0.0001, Mann-Whitney U test. Download FIG S3, TIF file, 0.4 MB. Copyright © 2019 Murer et al. 2019 Murer et al. This content is distributed under the terms of the Creative Commons Attribution 4.0 International license.

### Proliferation and LCL-specific cytokine production of CD8^+^ T cells are lower in ΔmiR than wt EBV-infected mice, whereas cytolytic granule contents of CD8^+^ T cells seem similar in the two infectious groups.

**FIG 4:**
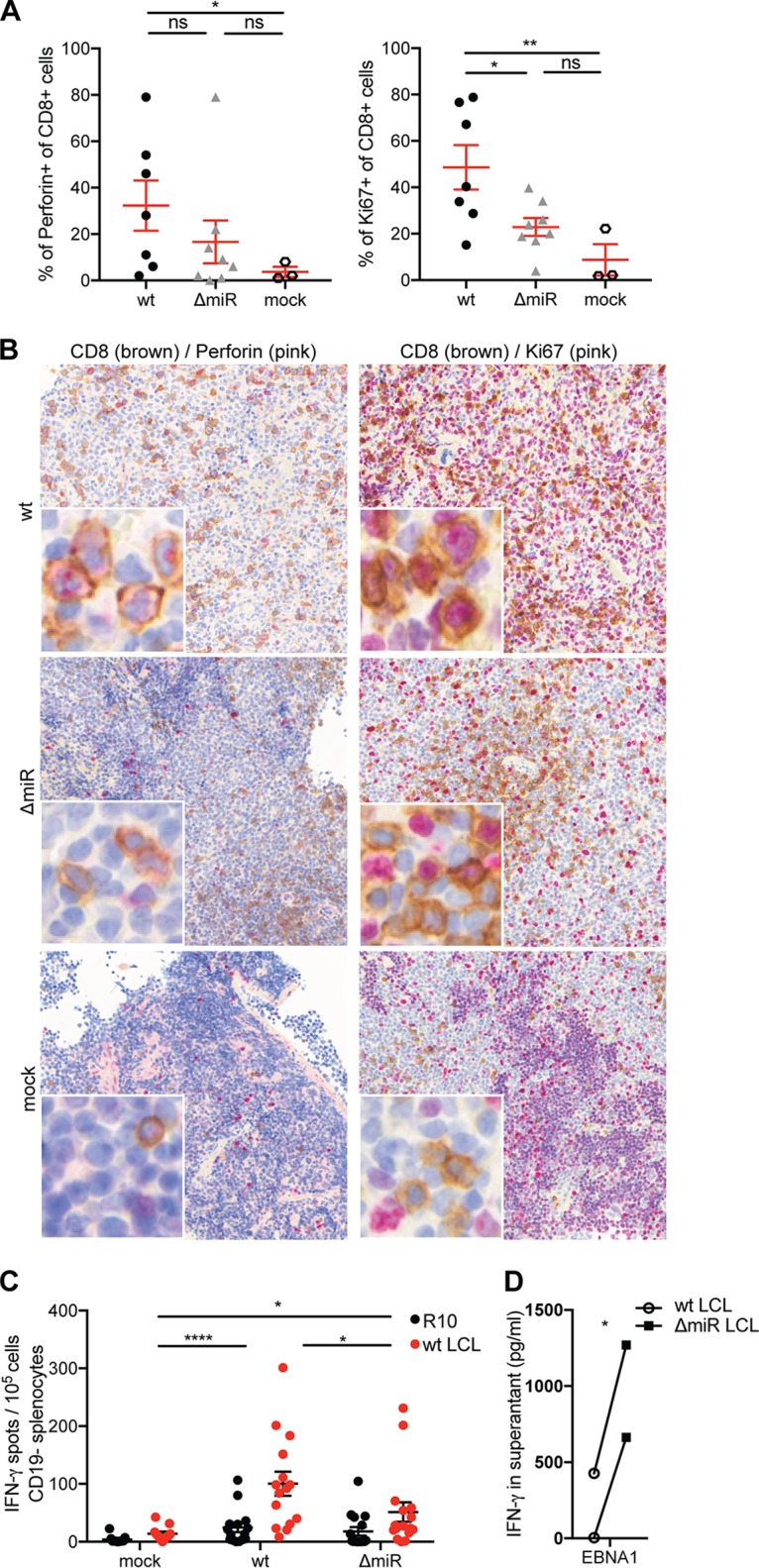
Proliferation and LCL-specific cytokine production of CD8^+^ T cells are lower in ΔmiR than wt EBV-infected mice, whereas cytolytic granule contents of CD8^+^ T cells seem similar in the two infectious groups. (A and B) Quantification of the frequency of perforin^+^ CD8^+^ cells of all CD8^+^ cells or Ki67^+^ CD8^+^ cells of all CD8^+^ cells (*n* = 3 to 8/group) (A) and the representative immunohistochemistry staining with CD8 (brown) and perforin (pink) (left column; original magnification, ×200) or CD8 (brown) and Ki67 (pink) (right column; original magnification, ×200) in splenic sections of huNSG mice infected with either ΔmiR or wt EBV 5 to 6 weeks p.i. or mock huNSG mice (B). (C) Evaluation of the EBV-specific T cell response through the measurement of the IFN-γ release via ELISpot assay upon coculture of CD19^−^ splenocytes, derived from ΔmiR or wt EBV-infected or noninfected (mock) huNSG mice, with autologous wt LCLs or medium (R10). (D) Assessment of the IFN-γ release via ELISA upon coculture of ΔmiR or wt LCL with an EBNA1-specific CD8^+^ T cell clone for 16 h. (A) Pooled data from 3 experiments represented with the mean ± SEM (*, *P* ≤ 0.05; **, *P* ≤ 0.01, unpaired *t* test with Welch’s correction). (C) Pooled data from 5 experiments represented with the mean ± SEM (*, *P* ≤ 0.05; ****, *P* ≤ 0.0001, Mann-Whitney U test). (D) Data from 2 experiments (*, *P* ≤ 0.05, paired *t* test).

To investigate the immune response to EBV more thoroughly, we examined Ki67 and perforin expression in CD8^+^ T cells on splenic sections of infected huNSG mice. The frequency of CD8^+^ T cells positive for the cytolytic effector molecule perforin was significantly increased in wt but not in ΔmiR EBV-infected mice compared to noninfected controls, whereas no significant difference between wt and ΔmiR EBV-infected mice was observed (Fig. 4A and B, left panels). Nevertheless, we detected a significantly higher frequency of proliferating CD8^+^ cells in wt EBV-infected than ΔmiR EBV-infected mice or noninfected controls (Fig. 4A and B, right panels). Additionally, we assessed EBV-specific T cell responses by coculturing CD19^−^ splenocytes with autologous LCLs generated with wt EBV and analyzed IFN-γ release by ELISpot assay. Cocultures containing splenocytes isolated from wt-infected mice showed significantly increased EBV-specific T cell reactivity compared to cocultures with splenocytes from ΔmiR-infected or mock-infected mice (Fig. 4C). However, splenocytes from ΔmiR-infected mice still produced significantly more IFN-γ spots than cells from noninfected mice (Fig. 4C). Thus, EBV-specific T cell responses were detected in all infected groups but were higher in wt than in ΔmiR EBV-infected mice. Nevertheless, as shown in a previous study (12), we found increased IFN-γ release when coculturing EBV-specific CD8^+^ T cell clones with autologous ΔmiR LCL compared to coculture with wt LCLs (Fig. 4D). This suggests that the less expanded CD8^+^ T cell compartment might control ΔmiR EBV-infected B cells more efficiently; even so, miRNA-deficient EBV infection induces less T cell proliferation, thereby accumulating fewer cytololytic granule-containing and cytokine-producing CD8^+^ T cells *in vivo*.

### CD8 depletion rescues impaired infectious capacity of EBV devoid of its miRNAs.

**FIG 5:**
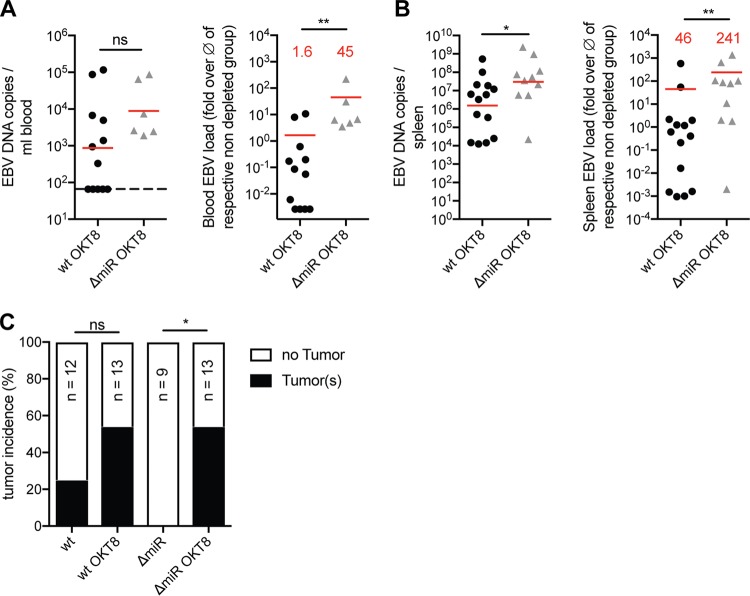
CD8 depletion rescues impaired viral infectious capacity of EBV devoid of its miRNAs. (A and B) Blood (A) and spleen (B) DNA viral loads determined by qPCR of huNSG mice (*n* = 5 to 14/group) infected with either wt or ΔmiR EBV and treated with OKT8 for CD8^+^ T cell depletion (left) or the matching relative viral loads normalized to the mean of the corresponding nondepleted groups (*n* = 8 to 12/group) with the indication of the fold difference (red) for the different infection groups (right) 5 weeks p.i. (A) Horizontal dashed line indicates the lower limit of quantification (LLOQ). Values below the LLOQ were raised to the LLOQ and plotted on the LLOQ line. (C) Frequency of tumor formation observed in mice infected with wt or ΔmiR EBV nontreated or treated with OKT8. (A and B) Pooled data from 4 experiments with geometric mean (left) and arithmetic mean (right). *, *P* ≤ 0.05; **, *P* ≤ 0.01, Mann-Whitney U test. (C) Pooled data from 4 experiments. *, *P* ≤ 0.05, Fisher’s exact test.

In order to address the T-cell-mediated immune control of ΔmiR EBV infection more specifically, we depleted CD8^+^ T cells with OKT8 antibodies before and during the infection of huNSG mice with wt or ΔmiR EBV. Depletion of CD8^+^ T cells led to similar or even increased EBV loads in ΔmiR EBV-infected compared to wt EBV-infected huNSG mice (Fig. 5A and B, left panels). Normalizing the viral loads to infected mice not treated with OKT8 antibodies revealed that the fold increases over the average of the matching nondepleted groups were significantly higher, up to 240-fold, in the ΔmiR- compared to wt-infected animals in blood and spleen (Fig. 5A and B, right panels). Hence, ΔmiR EBV seems to be better controlled by CD8^+^ T cells *in vivo* than wt EBV. Furthermore, although ΔmiR EBV infection in huNSG mice never induced tumor formation in the presence of an unmanipulated T cell compartment, upon OKT8 treatment tumor incidence reached levels comparable to wt EBV-infected mice (Fig. 5C). Thus, these data suggest that EBV miRNAs are able to reduce immune surveillance by T cells *in vivo*.

### Absence of CD8^+^ T cells alters CD4^+^ T cell recognition of miRNA-deficient EBV.

**FIG 6:**
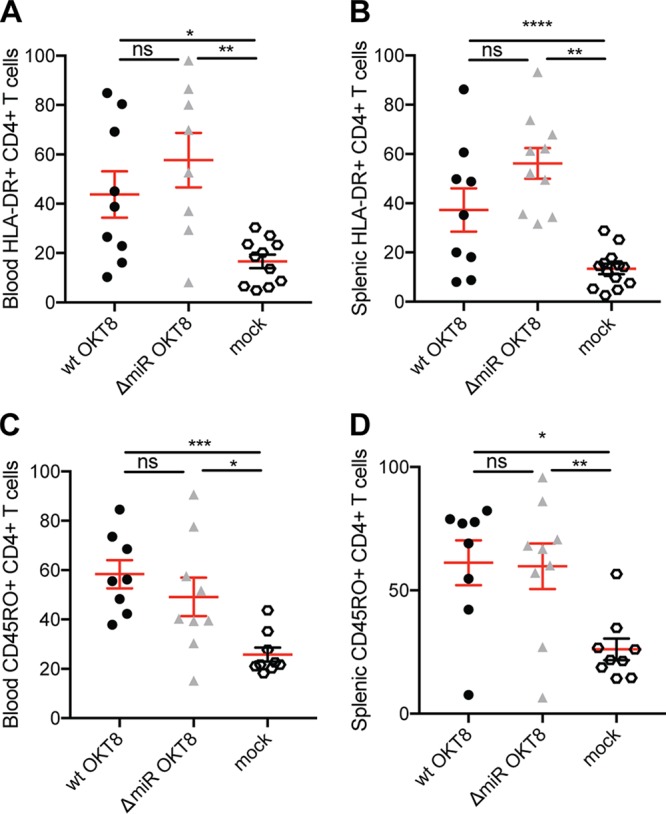
Absence of CD8^+^ T cells alters CD4^+^ T cell recognition of EBV without viral miRNAs. The frequencies of blood (A) and splenic (B) HLA-DR^+^ CD4^+^ T cells and of blood (C) and splenic (D) CD45RO^+^ CD4^+^ T cells of huNSG mice infected with either wt or ΔmiR EBV treated with OKT8 for CD8^+^ T cell depletion 5 weeks p.i. (*n* = 3 to 13/group) as determined by flow cytometry. (A to D) Pooled data from 2 to 3 experiments with mean ± SEM. *, *P* ≤ 0.05; **, *P* ≤ 0.01; ***, *P* ≤ 0.001; ****, *P* ≤ 0.0001, Mann-Whitney U test.

Since reduced frequencies of HLA-DR^+^ CD45RO^+^ T cells were observed in ΔmiR compared to wt EBV-infected huNSG mice, we aimed to investigate whether the absence of CD8^+^ T cells changed CD4^+^ T cell activation and memory formation. Specifically, we explored HLA-DR and CD45RO expression on CD4^+^ T cells. In both blood and spleen, there was a trend toward higher frequencies of activated HLA-DR^+^ CD4^+^ T cells in ΔmiR compared to wt EBV-infected mice treated with OKT8, though no significant differences were observed (Fig. 6A and B). However, the frequency of memory CD45RO^+^ CD4^+^ T cells seemed comparable between ΔmiR- and wt-infected mice inoculated with OKT8 (Fig. 6C and D). Nevertheless, we observed higher frequencies of blood and splenic HLA-DR^+^ CD4^+^ T cells (Fig. 6A and B) and higher frequencies of blood and splenic CD45RO^+^ CD4^+^ T cells in OKT8-treated wt and ΔmiR EBV-infected mice compared to noninfected mice (Fig. 6C and D). Thus, the immune response to ΔmiR EBV seems to generate a slightly higher activation and comparable memory formation in the remaining T cells when depleted for CD8^+^ T cells, suggesting that CD4^+^ T cells might also be more efficiently activated by ΔmiR EBV-infected cells.

## DISCUSSION

EBV was among the first viruses which were described to express miRNAs (21, 22). A wide spectrum of cellular, host, and viral functions have been described to be regulated by at least 44 mature miRNAs that EBV expresses (3, 23). Our *in vivo* model of EBV infection in huNSG mice now reveals that the main outcome of loss of miRNA expression is elevated T-cell-mediated immune control of viral infection and associated tumorigenesis. This improved immune control is associated with decreased numbers of proliferating EBV-infected B cells and attenuated T cell expansion. Consistent with a previous study that already demonstrated delayed kinetics of EBV infection and a tendency toward lower virus-associated lymphomagenesis in the absence of the BHRF1 miRNAs (14), we also find reduced viral loads and no detectable tumorigenesis with the complete miRNA-deficient virus but unchanged infection with BART miRNA knockout EBV. Furthermore, deletion of the EBERs, another prominent species of EBV-expressed nontranslated RNAs, has also no significant effect on EBV infection in huNSG mice (24). This suggests that the BHRF1 miRNAs influence viral infection and tumor formation more strongly than EBERs or the BART miRNAs, and this regulation mainly affects T cell recognition.

Immune evasion by miRNAs affects all stages of the EBV life cycle because it also covers the latent EBV infection programs that are important for the virus to persist, while most of the immune evasive proteins encoded by the virus are expressed only during lytic replication. These fall into four categories, inhibiting immune sensing via Toll-like receptors (TLRs) and the downstream NF-κB activation, compromising the type I IFN response, downregulating MHC-restricted antigen presentation, and changing a pro- into an anti-inflammatory environment (25). For example, BGLF5 and BPLF1 downregulate TLRs or interfere with TLR-mediated NF-κB activation (26, 27). Furthermore, BZLF1, BRLF1, BILF1, and BGLF4 reduce expression or inhibit the function of interferon-responsive factors (IRFs) 3 and 7 to compromise type I IFN responses (28–31). EBV also encodes BNLF2a, a protein that blocks peptide import into the endoplasmic reticulum (ER) via the transporter associated with antigen presentation (TAP) (32–35); BILF1, a protein that reduces MHC class I surface levels (34, 36, 37); BZLF2, a protein that interferes with T cell receptor binding to MHC class II (38, 39); and BDLF3, a protein that shields MHC class I- and II-restricted antigen presentation from T cell recognition (40). Finally, EBV-infected cells secrete a soluble macrophage colony-stimulating factor (M-CSF) receptor that is encoded in the BARF1 gene (41, 42) and viral IL-10 from the BCRF1 gene (43), which reduce inflammation and thereby immune surveillance of infected cells. In contrast, latent EBV proteins mainly diminish their own antigen presentation in *cis*, by limiting their protein expression and/or actively inhibiting their proteasomal degradation, as is the case for EBNA1 (44, 45). Thus, virally encoded proteins contribute to immune evasion in *trans* mainly during lytic EBV replication, while nontranslated miRNAs might primarily serve this purpose during latent EBV infection.

For this purpose, EBV miRNAs attenuate viral antigen production, compromise MHC class I-restricted antigen presentation via TAP downregulation and MHC class II-restricted antigen presentation via inhibition of lysosomal degradation, and diminish production of the T cell chemoattractant CXCL11 and the T-cell-priming cytokine IL-12 (3). For antigen recognition by CD8^+^ T cells, primarily TAP2 mRNA downregulation and targeting by the RNA-induced silencing complex (RISC) have been identified as viral miRNA targets (12). This diminishes also TAP1 protein levels as well as surface expression of especially HLA-B molecules, which are the main MHC class I restriction elements of immunodominant EBV-specific CD8^+^ T cell responses (46). Even though our T cell depletion experiments clearly argue that such miRNA functions dominate the outcome of EBV infection at week 5 with respect to viral loads and tumor formation, other previously *in vitro*-defined functions of EBV miRNAs might be responsible for the lower viral loads that we detect already at week 3 after infection. At this 3-week time point, lytic replication contributes to viral loads in our *in vivo* model of EBV infection, as could be demonstrated by comparing wild-type with lytic-replication-deficient (BZLF1 knockout) virus (16). Along these lines, sumoylation seems to be required for infectious virus production, and the SUMO ligase RNF4 is downregulated by the BHRF1-1 miRNA (47). This could suggest that diminished lytic EBV replication could contribute to the lower viral loads at week 3 after infection. Furthermore, BHRF1 miRNAs of EBV have been suggested to be required for efficient B cell transformation by attenuating viral protein expression (20, 48). They seem to optimize the expression timing of EBNA-LP and BHRF1, a viral antiapoptotic Bcl-2 homologue, in *cis*, when they get processed from the transcripts of these viral proteins that also contain their pri-miRNAs (49, 50). Also, these mechanisms might contribute to the diminished viral loads upon miRNA-deficient EBV infection in huNSG mice at early time points after infection. However, these defects seemed to be transient *in vitro*, and no growth defects could be detected in established EBV-transformed B cell lines. Similarly, in our studies at 5 weeks postinfection miRNA-mediated attenuation of T cell responses predominated and viral loads as well as tumor formation by ΔmiR EBV could be restored by T cell depletion. Thus, miRNA deficiency could be further explored to promote immune responses with an attenuated EBV that might be considered for prophylactic vaccination against symptomatic primary infection, i.e., infectious mononucleosis.

## MATERIALS AND METHODS

### Recombinant EBV.

Concentrates of the green fluorescent protein (GFP)-encoding Epstein-Barr virus (EBV) miRNA knockout (ΔmiR, p4027), EBV BART miRNA knockout (ΔmiR-BART, p5446), or EBV wild-type (wt, p2089) viruses were prepared as preciously described (12, 13, 51) and titrated on Raji cells. The miRNA-expressing loci in the ΔmiR and ΔmiR-BART viruses were replaced with computed scrambled sequences as described previously (15). The GFP-expressing cells were quantified by flow cytometry 2 days later to calculate Raji-infecting units (RIU). For *in vitro* infection of B cells, B cells isolated from splenocytes or human PBMCs were isolated using anti-CD19 microbeads (Miltenyi Biotec) and infected with wt or ΔmiR EBV with the addition of a CD40L-expressing feeder cell line irradiated with 140 Gy. The cells were maintained in RPMI 1640 medium (Gibco, Thermo Fisher Scientific) with 10% FCS, streptomycin, and penicillin in the absence of the CD40L-expressing feeder cell line. Total numbers of established LCLs were determined by trypan blue staining.

### Mice with reconstituted human immune system components and infection with EBV.

NOD-*scid* γ_c_^null^ (NSG) mice and HLA-A2 transgenic NSG mice were obtained from the Jackson Laboratories and maintained under specific-pathogen-free conditions. Reconstitution was performed as previously described (52). Reconstitution level was checked 10 to 12 weeks later and again a week prior to the start of the experiments by flow cytometric immune phenotyping of peripheral blood as previously described (16). Mice were infected intraperitoneally with 1 × 10^5^ RIU of wt, ΔmiR, or ΔmiR-BART EBV and followed for 5 or 6 weeks. Samples below the lower limit of quantification (LLOQ) of 67 EBV DNA copies were considered negative for EBV DNA. The value of the LLOQ was used for the blood DNA copies of mice having a viral load in the spleen but not in the blood. Mice without any viral loads in the blood and spleen throughout the experiment were considered not infected and excluded from the analysis.

### Purification of mouse anti-human CD8 antibody.

OKT8 hybridoma cells (mouse IgG2a; ATCC, Manassas, VA, USA) were gradually adapted to serum- and protein-free PFHM-II medium (Thermo Fisher Scientific [formerly Gibco], catalog no. 12040-077). Cells were cultured in BD CELLine 1000 flasks (BD catalog no. 353137) according to the manufacturer’s recommendation. Antibodies were precipitated from the culture supernatant by addition of an equal volume of saturated ammonium-sulfate solution. After buffer exchange to PBS using PD-10 desalting columns (Sephadex G25 medium; GE Healthcare), antibodies were sterilized by filtration through 0.2-µm filters (Filtropur S 0.2; Sarstedt) and stored at 4°C until use.

### Depletion of T cells.

Human CD8^+^ T cells were depleted in mice before EBV infection by intraperitoneal injection of 100 µg OKT8 antibodies on three consecutive days. To deplete T cells for the duration of the experiment, 50 µg of the depletion antibodies was injected 3 times a week starting 2 weeks postinfection until the day of sacrifice.

### Quantification of viral load.

DNA from splenic tissue was obtained using a QIAamp DNA tissue kit (Qiagen) and from whole blood using the NucliSENS EasyMAG System (bioMérieux), both according to the manufacturers’ recommendations. The TaqMan (Applied Biosystems) real-time PCR technique was performed to quantitatively analyze EBV DNA as previously described (53), with modified primers for the BamHI W fragment (5′-CTTCTCAGTCCAGCGCGTTT-3′ and 5′-CAGTGGTCCCCCTCCCTAGA-3′) and a fluorogenic probe (5′-FAM-CGTAAGCCAGACAGCAGCCAATTGTCAG-TAMRA-3′). All PCRs were run on an ABI Prism 7300 Sequence Detector or ViiA7-240 Real-Time PCR system (Applied Biosystems), and samples were analyzed in duplicate.

### Flow cytometry.

All fluorescently labeled antibodies are listed in Table S1 in the supplemental material. Lysis of erythrocytes in whole blood was done with NH_4_Cl. Spleens were mashed and filtered through a 70-μm cell strainer before separation of mononuclear cells on Ficoll-Paque gradients. Cell suspensions were stained with antibodies for 30 min at 4°C, washed, and analyzed on FACS Canto or LSR Fortessa cytometers (BD Biosciences). Analysis of flow cytometric data was performed with FlowJo (Tree Star).

10.1128/mBio.01941-18.4 TABLE S1 Overview of fluorescently labeled antibodies for flow cytometry. Download Table S1, DOCX file, 0.1 MB. Copyright © 2019 Murer et al. 2019 Murer et al. This content is distributed under the terms of the Creative Commons Attribution 4.0 International license.

### Histology, immunohistochemistry, and immunofluorescence.

Tissue was fixed in 10% saline-buffered formalin and paraffin embedded. For immunohistochemistry and immunofluorescence, 3-μm sections were processed on a Leica Bond-Max or Bond-III automated immunohistochemistry system. Stainings were performed with monoclonal mouse anti-EBNA2 (clone PE2; Abcam), rabbit anti-Ki67 (clone SP6; Cell Marque), rabbit anti-huCD8 (clone SP16; Marque), mouse antiperforin (5B10; Novocastra Laboratories), and polyclonal cleaved caspase 3 (Cell Signaling Technology). For immunofluorescence, the AF488 donkey anti-rabbit IgG (Jackson ImmunoResearch), DyLight549 horse anti-mouse IgG (Vectrolabs), and DAPI (Sigma) were used. Stainings were analyzed as described elsewhere (52) using the Vectra3 automated quantitative pathology imaging system (PerkinElmer) and InForm software analysis (PerkinElmer). The percentage of EBNA2 plus Ki67, EBNA2 plus cleaved caspase 3, huCD8 plus Ki67, or huCD8 plus perforin double-positive cells of all EBNA2- or huCD8-positive cells was calculated.

### EBV-specific IFN-γ release assay (ELISpot).

EBV-specific T cell responses were analyzed using an IFN-γ enzyme-linked immunospot (ELISpot) assay as previously described (10, 54). Briefly, splenocytes were depleted of human CD19^+^ cells using anti-CD19 microbeads (Miltenyi Biotec). The CD19-depleted fraction was stimulated with autologous LCLs at a ratio of 1:4 for 18 h, and incubation with R10 and phorbol myristate acetate (PMA)-ionomycin served as negative and positive controls, respectively. Each condition was performed in duplicates. Spots were counted on an ELISpot reader system (ELR02; Autoimmun Diagnostika GmbH).

### CD8^+^ T cell cloning and restimulation.

CD8^+^ T cell clones specific for the nuclear antigen 1 of EBV (EBNA1) were generated from freshly isolated PBMCs which were depleted of CD4^+^ T cells using anti-CD4-conjugated MACS microbeads and magnetic selection (Miltenyi Biotec). PBMCs were stimulated with 5 μM EBNA1-derived HPVGEADYFEY peptide (aa 407 to 417) for 3 to 4 h. Responding cells were enriched by IFN-γ secretion assay (130-054-201; Miltenyi Biotec) and were cloned by limiting dilution at 3 and 30 cells per well on HLA-matched irradiated LCLs (10^4^/well) loaded with the relevant peptide at 5 μM and allogeneic irradiated, PHA-treated PBMCs (10^5^/well) in medium with 10% pooled human sera supplemented with 150 IU/ml IL-2 (T cell medium). Growing microcultures were screened for peptide reactivity by IFN-γ ELISA (3420-1H-20; Mabtech). Selected CD8^+^ T cell clones were further expanded on autologous irradiated LCLs loaded with the relevant peptide at 5 μM and allogeneic irradiated, PHA-treated PBMCs in T cell medium. Clones were maintained in T cell medium. Every 2 to 3 weeks, CD8^+^ T cell clones were restimulated with peptide-pulsed autologous LCLs and allogeneic PHA-treated PBMCs. The resulting CD8^+^ T cell clone used in this study was specific for EBNA1_407–417_ (HPVGEADYFEY, HLA-B*3501). To investigate specific recognition of target cells, 10,000 CD8^+^ T cell clones were cocultured with 100,000 autologous wt or ΔmiR LCLs. After overnight incubation, coculture supernatants from single wells were tested by ELISA for IFN-γ content.

### Statistical analysis.

Statistical analysis and graph preparation were performed with Prism software (GraphPad). A paired *t* test, unpaired *t* test with Welch’s correction, or two-tailed Mann-Whitney U test was used. A *P* value of <0.05 was considered statistically significant.

### Study approval.

All animal protocols were approved by the cantonal veterinary office of the canton of Zürich, Switzerland (protocols 209/2014 and 159/2017). All studies involving human samples were reviewed and approved by the cantonal ethics committee of Zürich, Switzerland (protocol KEK-StV-Nr.19/08).
