# Erratum for Murer et al., “MicroRNAs of Epstein-Barr Virus Attenuate T-Cell-Mediated Immune Control *In Vivo*”

**DOI:** 10.1128/mBio.01482-19

**Published:** 2019-07-23

**Authors:** Anita Murer, Julia Rühl, Andrea Zbinden, Riccarda Capaul, Wolfgang Hammerschmidt, Obinna Chijioke, Christian Münz

**Affiliations:** a Viral Immunobiology, Institute of Experimental Immunology, University of Zürich, Zürich, Switzerland; b Institute of Medical Virology, University of Zürich, Zürich, Switzerland; c Research Unit Gene Vectors, Helmholtz Zentrum München, German Research Center for Environmental Health and German Centre for Infection Research (DZIF), Partner Site Munich, Munich, Germany; d Institute of Pathology and Medical Genetics, University Hospital Basel, Basel, Switzerland

## ERRATUM

Volume 10, no. 1, e01941-18, 2019, https://doi.org/10.1128/mBio.01941-18. After careful review of Fig. 3, it was noted that for the middle panels of Fig. 3C and D *P* values instead of *r* values had been reported. The revised Fig. 3 shown here reports the corrected *r* values for the correlations of ΔmiR EBV DNA copies/spleen with the percentage of splenic HLA-DR^+^ CD45RO^+^ CD4^+^ T cells (Fig. 3C) and with the percentage of splenic HLA-DR^+^ CD45RO^+^ CD8^+^ T cells (Fig. 3D).

**FIG 3:**
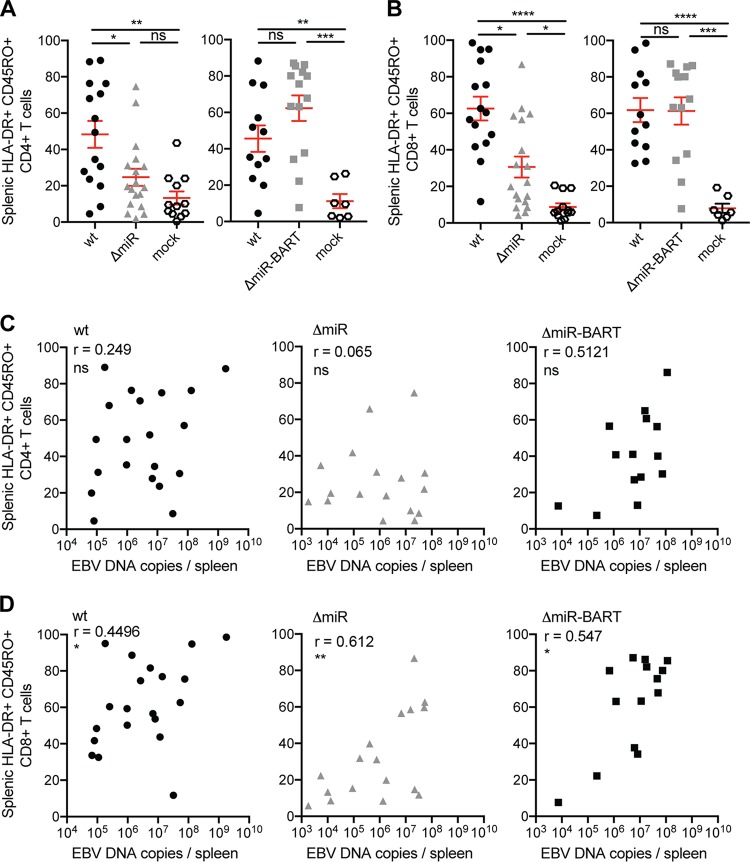
Activation and memory formation of CD8^+^ T cells correlate with EBV viral load. (A and B) The frequency of splenic HLA-DR^+^ CD45RO^+^ CD4^+^ T cells (A) and splenic HLA-DR^+^ CD45RO^+^ CD8^+^ T cells (B) of huNSG mice infected with either 10^5^ RIU of wt, ΔmiR, or ΔmiR-BART EBV 5 to 7 weeks p.i. or mock huNSG mice (n = 7 to 18/group) was determined by flow cytometry. (C and D) Correlation of the frequencies of activated memory CD4^+^ (C) and activated memory CD8^+^ (D) T cells, from panels A and B, respectively, with the splenic endpoint viral DNA loads as determined by qPCR for each infected group. (A and B) Pooled data from 4 wt and ΔmiR-BART and 5 wt and ΔmiR experiments with mean ± SEM. *, *P* ≤ 0.05; **, *P* ≤ 0.01; ***, *P* ≤ 0.001; ****, *P* ≤ 0.0001, Mann-Whitney U test. (C and D) Pooled data from 4 to 7 experiments. *, *P* ≤ 0.05; **, *P* ≤ 0.01, Spearman correlation.

