# Supplementary figures and images for: MicroRNAs of Epstein-Barr Virus Attenuate T-Cell-Mediated Immune Control In Vivo
Source: mBio. 2019 Jan 15;10(1):e01941-18. doi: 10.1128/mBio.01941-18 (PMC6336420; doi:10.1128/mBio.01941-18)

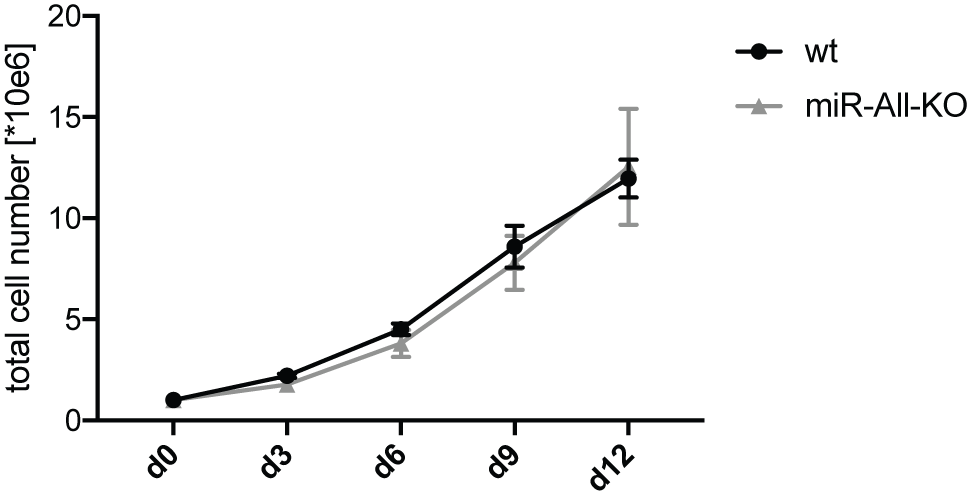

Supplement: FIG S1 [file mBio.01941-18-sf001.tif]

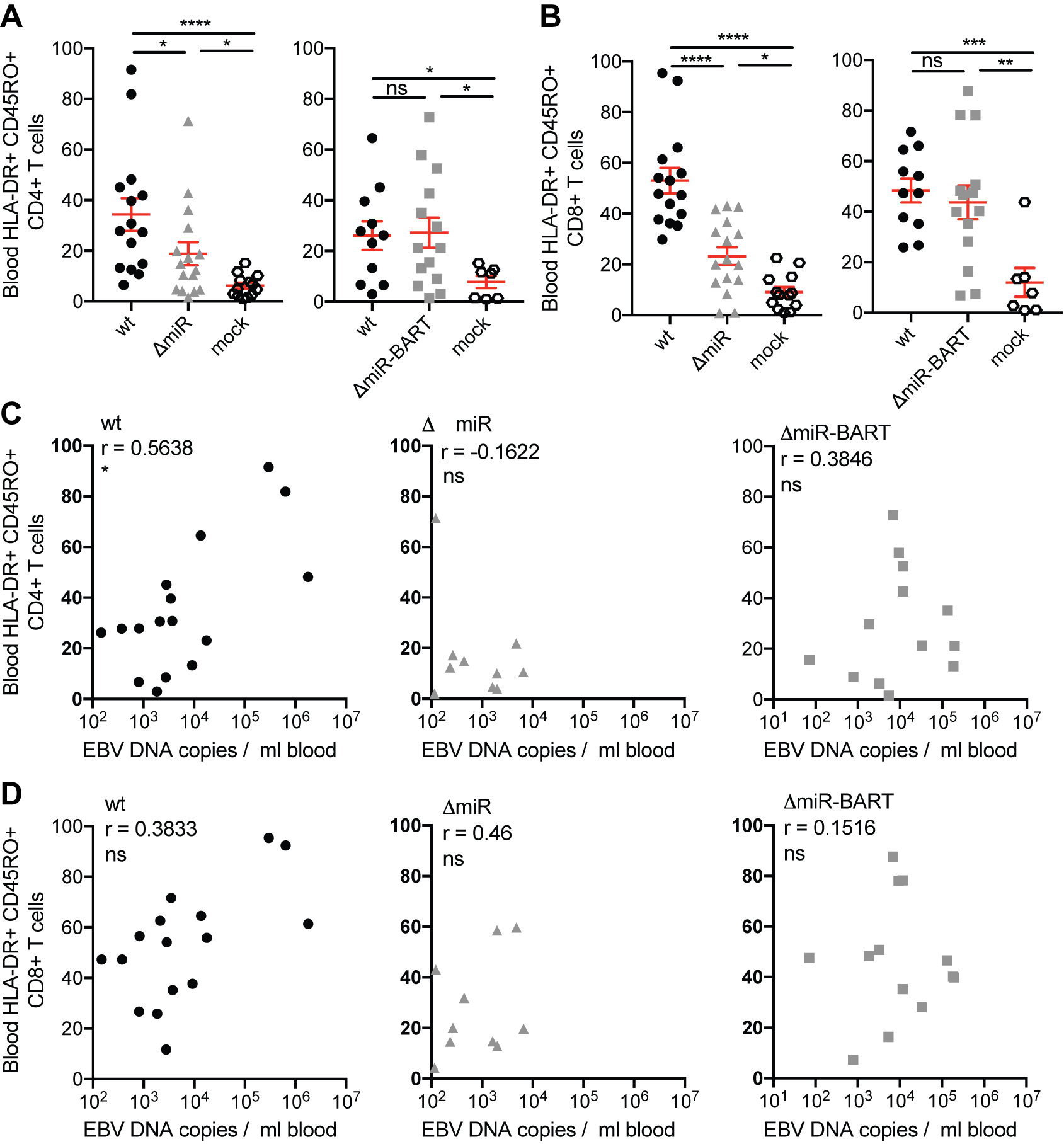

Supplement: FIG S2 [file mBio.01941-18-sf002.tif]

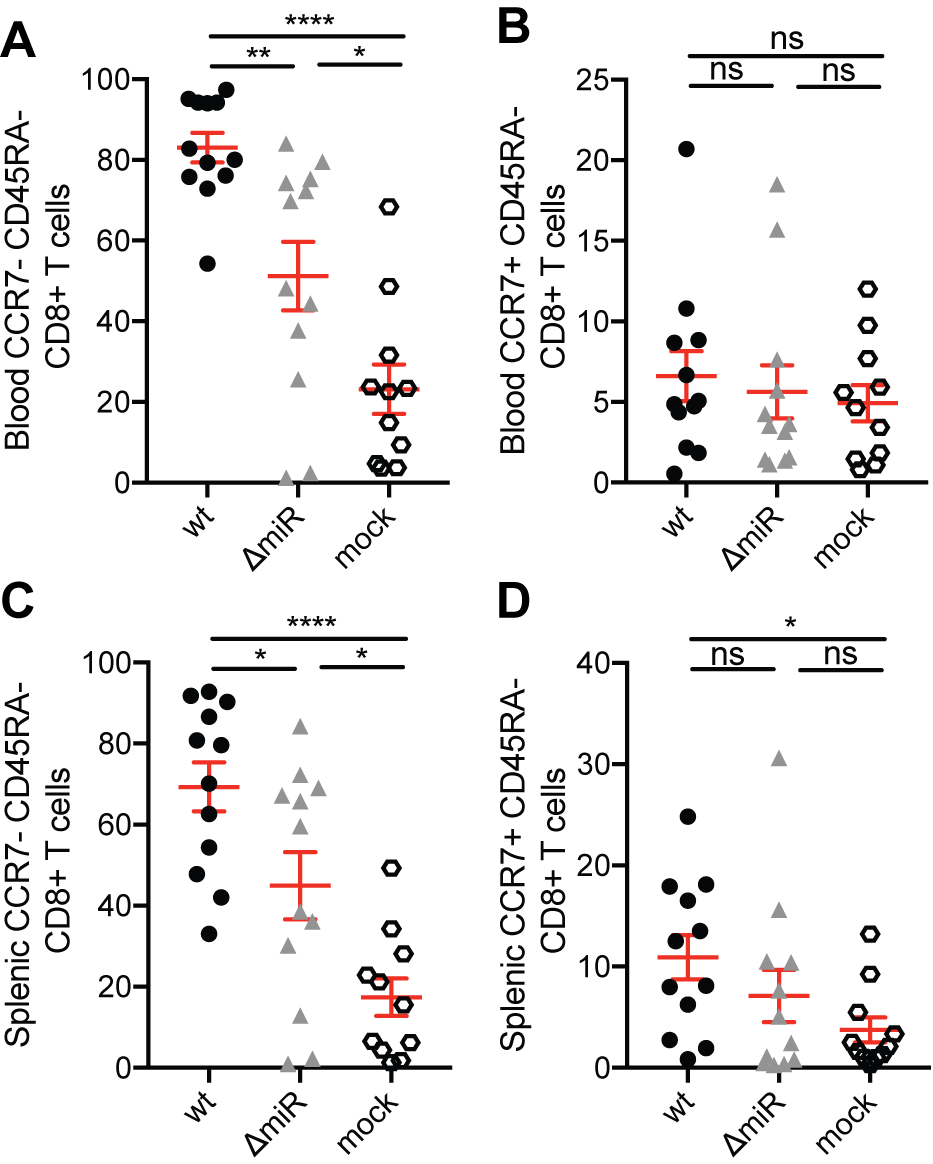

Supplement: FIG S3 [file mBio.01941-18-sf003.tif]
