# Supplementary material for: MicroRNAs of Epstein-Barr Virus Attenuate T-Cell-Mediated Immune Control In Vivo
Source: mBio. 2019 Jan 15;10(1):e01941-18. doi: 10.1128/mBio.01941-18 (PMC6336420; doi:10.1128/mBio.01941-18)
Supplement: TABLE S1 [file mBio.01941-18-st001.docx]

**Table S1. Overview of fluorescently labeled antibodies for flow cytometry**

| Molecule | Clone | Fluorophore | Company |
| --- | --- | --- | --- |
| NKp46 | 9E2 | APC | BD Bioscience |
| CD19 | HIB19 | PE-Cy7 | Biolegend |
| CD3 | VCHT1 | PE | Biolegend |
| CD3 | OKT3 | BV785 | Biolegend |
| CD4 | RPA-T4 | APC-Cy7 | Biolegend |
| CD4 | RPA-T4 | BV510 | Biolegend |
| CD45 | HI30 | Pacific Blue | Biolegend |
| CD45 | HI30 | BV605 | Biolegend |
| CD45RA | HI100 | BV510 | BioLegend |
| CD45RO | UCHL1 | AlexaFluor700 | Biolegend |
| CD8 | SK1 | PerCP | Biolegend |
| HLA-DR | L243 | FITC | Biolegend |
| HLA-DR | L243 | Pe-Cy7 | Biolegend |
| CD19 | SJ25-C1 | PE Texas Red | Invitrogen |
| CCR7 | 150503 | PE | R&D Systems |
